# Cluster Sampling Bias in Government-Sponsored Evaluations: A Correlational Study of Employment and Welfare Pilots in England

**DOI:** 10.1371/journal.pone.0160652

**Published:** 2016-08-09

**Authors:** Arnaud Vaganay

**Affiliations:** London School of Economics and Political Science, London, United Kingdom; University of Reading, UNITED KINGDOM

## Abstract

For pilot or experimental employment programme results to apply beyond their test bed, researchers must select ‘clusters’ (i.e. the job centres delivering the new intervention) that are reasonably representative of the whole territory. More specifically, this requirement must account for conditions that could artificially inflate the effect of a programme, such as the fluidity of the local labour market or the performance of the local job centre. Failure to achieve representativeness results in Cluster Sampling Bias (CSB). This paper makes three contributions to the literature. Theoretically, it approaches the notion of CSB as a human behaviour. It offers a comprehensive theory, whereby researchers with limited resources and conflicting priorities tend to oversample ‘effect-enhancing’ clusters when piloting a new intervention. Methodologically, it advocates for a ‘narrow and deep’ scope, as opposed to the ‘wide and shallow’ scope, which has prevailed so far. The PILOT-2 dataset was developed to test this idea. Empirically, it provides evidence on the prevalence of CSB. In conditions similar to the PILOT-2 case study, investigators (1) do not sample clusters with a view to maximise generalisability; (2) do not oversample ‘effect-enhancing’ clusters; (3) consistently oversample some clusters, including those with higher-than-average client caseloads; and (4) report their sampling decisions in an inconsistent and generally poor manner. In conclusion, although CSB is prevalent, it is still unclear whether it is intentional and meant to mislead stakeholders about the expected effect of the intervention or due to higher-level constraints or other considerations.

## Introduction

Cluster sampling is frequent in applied research. It is particularly relevant when sampling frames are not readily available or when the target population is widely dispersed geographically, making both service provision and data collection costs relatively high. Typical clusters include hospitals, schools, employment agencies, police areas, tribunals, etc. It is through these clusters that patients, pupils, jobseekers or victims of crime are recruited for a given clinical trial or a social experiment. It is also based on these clusters that inferences are made about the effect of a treatment or intervention in the population of interest.

*Cluster sampling bias* (CSB) is a type of sampling bias specific to cluster sampling. It occurs when some clusters in a given territory are more likely to be sampled than others. It is related to–but distinct from–*subject sampling bias*, which occurs when individuals sharing a specific characteristic (e.g. a similar socio-economic background or health status) are oversampled. This latter type of bias is not discussed here. Regardless of whether it occurs at cluster or subject level, sampling bias can be alleviated by using probability sampling methods and larger samples. This can be difficult to achieve in applied research, where limited resources and conflicting priorities often lead investigators to make decisions that are ‘good enough’ rather than scientifically ‘optimal’ [1]–[2]. The prevalence of these constraints suggests that sampling bias is common in applied research [3]–[4].

Whether the prevalence of CSB ought to concern researchers and policy-makers is a different matter. Not all cluster characteristics–and thus not all sampling biases–are policy-relevant; however, many are. These include variations in terms of local context (e.g. a given hospital can be located in a relatively younger or more affluent region) and in terms of their practice and performance (e.g. some schools achieve better results than others). These variations can, and often do, affect the effectiveness of the treatment or intervention being evaluated [5]. When such sample biases occur, stakeholders can be misled by the implied effectiveness of the intervention on a national scale.

Although the term ‘cluster sampling bias’ is infrequently used (no reference on the Web of Science and seven references on Google as of May 2015), the issue is well documented in the literature, mainly as an example of a threat to external validity. Findings of CSB have often been serendipitous: for example, the evaluation of Charter Schools in the US found school-specific impacts that varied from significantly negative to significantly positive [6]. Bloom and Weiland have found equally striking, and statistically significant, variations in impacts on various outcomes in the National Head Start Study [7]. Recent years have seen increasing interest in the question of external validity *per se*, often through opinion pieces and reflective papers [3]–[8]–[9]. This, in turn, has triggered a series of studies looking at the prevalence of the problem [4]–[10]–[11]. A conceptual model of purposive site selection has been developed [4]. Corrective and preventive measures have been formulated [3]–[12]. Over the past few years, external validity has moved from ‘secondary to internal validity’ to “an important area for scientific definition and investigation” [13] and even “one of the major flaws” of impact evaluations [3]–[8]–[14]. Yet, our knowledge of the phenomenon remains surprisingly superficial, not least because the problem is rarely reported and discussed in evaluation studies [15].

This study makes three main contributions. Theoretically, it approaches the notion of CSB not just as a statistical property, as in the above-mentioned studies, but as a human behaviour. The benefit of such an approach goes beyond scholarly discussions. Once causes and effects are identified, solutions (‘nudges’) can be developed, implemented, and evaluated. Unfortunately, the correlational design of this study did not allow testing the CSB theory in full. Nevertheless, I believe this is still a useful development. With this paper, I am hoping to trigger a discussion among stakeholders.

Methodologically, this study contributes to the literature by taking a ‘narrow and deep’ approach, which contrasts with ‘broad and shallow’ reviews of clinical trials undertaken so far and with anecdotal evidence that commonly exists in social research. I developed a new dataset for that purpose, using data systematically collected from 68 pilot evaluations commissioned by the UK Department for Work and Pensions (DWP) between 1997 and 2010. This creates a ‘narrow’ approach. The studies were systematically selected from the DWP’s research catalogue and steps were taken to minimize publication bias. The content of these studies was then screened to report which of the 40 Jobcentre Plus (JCP) districts had been selected as pilot areas. Binary logistic regression was used to model the odds of a district being selected as pilot site, controlling for a large number of policy-specific and area-specific variables. These measures constitute a ‘deep’ approach.

Empirically, this study was designed to address three questions: (1) Were pilot sites sampled with a view to maximise generalisability? (2) Did all sites have the same probability of being sampled? (3) Were ‘effect-enhancing’ clusters more likely to be sampled? A fourth and more exploratory question about the possible association between client caseload and probability of being sampled was added during the data analysis.

## Theoretical Framework

The fundamental idea that lies at the core of this study is that CSB is more than a statistical property; it is a human behaviour. This implies that statistical tools will only correct the problem if they are complemented by appropriate behavioural interventions. Given the relative novelty of this theory, it is imperative to begin with a definition.

### Effect of CSB

CSB results in the selection of a set of clusters that is not representative of the territory where the intervention is meant to be rolled out. This is far from a rare occurrence. Of the 273 randomized trials described in the *Digest of Social Experiments* [16], and reviewed by Olsen and colleagues, only seven were designed to be representative of the population of interest. According to the authors, this number has not increased by much since the Digest [4]. This insight confirms a previous finding that centres participating in clinical trials are rarely sampled with a view to maximise generalisability [10]–[11]–[17].

This does not necessarily mean that the conclusions of the corresponding evaluations will also be biased. If an intervention is expected to have the same impact everywhere, CSB is not relevant [4]–[5]. In practice, however, there is a high risk that the effect of an intervention be site-specific. Reviews of clinical trials have shown that the choice of participating centres often influence the generalisability of trial results [10]. Factors like hospital volume [18], practitioners’ expertise [19], and previous record of success [10] have all been shown to influence clinical outcomes. Similar observations have been made about the role of schools and children centres in social interventions [6]–[7].

In theory, CSB can result in *underestimating* the effect of the intervention. However, in a context where the boundary between research and development is increasingly tenuous, such an outcome might be sanctioned financially, politically and, in a way, scientifically (through the rejection by journal editors or papers failing to show statistically significant results). Thus, the CSB theory posits that the effect of an intervention is more likely to be *overestimated*. As an illustration, a meta-analysis of 46 surgical case series that published operative risks during the five years after the Asymptomatic Carotid Artery Study (ACAS) trial found operative mortality to be eight times higher and the risk of stroke and death to be about three times higher than in the original study [10]–[20]. To the best of my knowledge, no such evidence exists in social research.

At this stage, the riddle is almost solved. As most readers will have guessed by now, the simplest way one goes from the selection of a non-representative sample to a result that will be perceived by stakeholders as ‘favourable’ is through the hand-picking of ‘effect-enhancing’ clusters. There is some evidence that this might be the reality. For example, the afore-mentioned ACAS trial only accepted surgeons with an excellent safety record, rejecting 40% of applicants initially, and subsequently barring those who had adverse operative outcomes from further participation. The benefit from surgery in ACAS was due in major part to the consequently low operative risk [10]–[20]. There has been no systematic research so far into the external validity of the samples used in social policy evaluation. However, there is suspicion that the pilot sites used in social policy evaluation are exemplary rather than representative [21]–[22]–[23]–[24]–[25].

Such a scenario would be implausible if the issue of CSB was highly salient within the scientific community and if investigators were required to fully report their cluster sampling decisions. Yet, this is a far cry from the current reality [4]–[10]-–[15]–[26].

### Causes of CSB

Four reasons explain why a set of clusters might not be representative. The first cause includes the institutional or organisational constraints, over which the investigator has little or no control. These features will at times clash with the research objectives and mission and can make it very difficult to obtain a truly representative sample. A foremost example of this is the European Carotid Surgery Trial (ECST), mentioned by Rothwell [10]. In this study, there were national differences in the speed with which patients were investigated, with a median delay from last symptoms to randomisation of greater than two months in the UK, for example, compared with three weeks in Belgium and Holland. Separate trials in these systems would have produced very different results, because of the narrow time window for prevention of stroke [10]. A related problem is that of limited resources: researchers who cannot afford a larger sample may compromise by using a smaller one.

The external validity of a study might be compromised because of stakeholders’ interest or resistance. Sites are almost always allowed to opt out of participating. Greenberg and Barnow cite the example of the National Job Training Partnership Act Study. The evaluation was limited to those self-selected sites that were willing to participate. Conversely, the sites that consent to be part of a trial may be quite different from the types of clusters that would implement [8].

The third cause is a scientific interest in considering a target population somewhat different from the population directly targeted by the trial. This is the case with *efficacy trials* for example. Efficacy trials are typically designed to assess whether the treatment/intervention produces the expected result under ideal circumstances. Therefore, efficacy trials are not designed to produce results that are generalisable to any population of policy interest. In that sense, they differ from *effectiveness trials* which measure the treatment’s effect under ‘real world’ settings [27]. Efficacy and effectiveness exist on a continuum. Generalisability depends largely on the viewpoint of the observer and the condition under investigation [28].

Finally, sampling bias might result from cognitive dissonance. Researchers, like all human beings, uphold different values, and some of these values will sometimes be in conflict [29]. For example, researchers are required to strictly observe the norms of science. However, they might also have preferences for some pre-determined conclusions that they find morally ‘right’ or that confirm findings from previous studies. The tension between these different values and norms often result in psychological distress and in biased decisions. Some have argued that these biased decisions were often the product of automatic, unintentional strategy [30]–[31]. Others have retorted that it was a form of misconduct [32]–[33]. Importantly for the rest of this paper, confirmation bias is likely to be stronger when the issue is salient [34].

## The Case at Hand

To analyse CSB empirically, I developed a new dataset. The ‘PILOT-2’ dataset focuses on the employment pilots commissioned in England by the Department for Work and Pensions and its predecessors (Department for Social Security, Department for Employment and Skills) between May 1997 and May 2010. This period corresponds to the Labour governments of Tony Blair and Gordon Brown and was chosen primarily for its convenience. Considerations behind the selection of the place, time and policy area included: (a) a sufficient number of pilot studies to allow for robust statistical analyses; (b) a high degree of transparency in terms of publication and reporting; (c) the use of comparable areas across studies; and (d) the availability of data for each area.

The ‘case study’ approach to this paper results in limited generalisability. Results were triangulated with qualitative descriptions of the main actors, their motivations and constraints. This section briefly summarises the most salient points of this literature. Readers interested in a more detailed account are referred to a DWP-commissioned study describing the impact of research on the policy process [35], a *Science and Analysis Capability Reviews* of the DWP commissioned by the Government’s Office for Science (GO Science) [36], and an ethnographic study of the use of evidence in policy-making in the UK [37].

### Influence of policy commitments

An institutional analysis of government-sponsored research in the UK suggests that the research decisions made by DWP officials might be skewed towards preferred policy outcomes. The DWP is indeed a ministerial department, which is led politically by a government minister and covers matters that require direct political oversight, such as the formulation and implementation of new policies. In contrast, non-departmental public bodies (NDPBs) generally cover matters for which direct political oversight is judged unnecessary or inappropriate. Research Councils like the Medical Research Council or the Economic and Social Research Council are examples of NDPBs in the UK.

Unsurprisingly, ministerial departments like the DWP are organised to facilitate top-down policy delivery rather than science-driven policy formulation. This is evident in the DWP’s budget: in the late 2000s, the department spent an average of £20 million per year on ‘external research’ [36]. This amount must be compared with the DWP’s departmental expenditure limit, i.e. the budget allocated for the running of the services that it oversees and the cost of staff–which was £8.3 billion in 2012–2013. It is also evident in the DWP’s workforce: in 2011, 679 people out of the 100,000 staff employed by the DWP (including Jobcentre Plus) worked in policy research.

There is evidence that in the context of UK ministerial departments, the selection of pilot sites is made by ‘policy teams’ that are primarily responsible for the implementation of a policy reform. Although ‘analytical’ teams can and do provide input, they do not have the authority to make the formal decision [35]. Pilot sites in the DWP are chosen by policy teams with input from researchers. These teams are more concerned with the seamless implementation of reforms rather than the scientific quality of evaluations [35].

### Influence of research norms

Ethnographic accounts of the DWP paint a somewhat different picture: that of a department exceptionally committed to research and evidence-based policy. In 2011, GO Science conducted a *Science and Analysis Capability Review* of the DWP. The reviewers praised the “strong commitment across the Department to using analytical and scientific evidence to inform the development and delivery of policy”. They found that the focus on evidence was supported by the presence of economists and scientists in several senior policy delivery roles. The reviewers also found “consistently high levels of enthusiasm, commitment and retention among analytical staff which reflects and helps to perpetuate the focus on use of science and analysis” [36]. A Report from the National Audit Office indicated that eight out of ten labour market evaluations conducted by the DWP were of a sufficient standard to have confidence in the impacts attributed to the policy. This proportion was the highest among four policy areas [38].

This strong focus on science transpires in the selection of pilot sites–to a certain extent. Boa et al. [35] cite the evaluation of the *Pathways to Work* pilot, in which DWP analysts successfully got the pilot redesigned so that the evaluations provide more meaningful data. They indicate that, having made a convincing case, the size of the pilot doubled from three to seven areas.

More surprisingly perhaps, even the members of the Social Security Parliamentary Committee seemed to be concerned by issues of representativeness. The following extract from the Committee’s verbatim is telling:

“We understand the reasons why the present pilot areas were chosen, but the Government will need to bear in mind during the evaluation the fact that the pilot areas are not fully representative of the country as a whole. We recommend that, even at this late stage, the Government should give consideration to adding a pilot area which covers a predominantly London area or Northern city geographical type.” [39].

The Parliament’s recommendation prompted the following response from the DWP:

“We are confident that the pilot areas are sufficiently representative of the country as a whole for us to make sound estimates of the national impact of ONE. The selection of the pilot areas was determined primarily by the need to ensure that the pilots covered a range of labour markets and demographic characteristics, and the areas selected (such as Lea Roding and Leeds) include characteristics of concern to the Committees such as deprivation and representation of ethnic minorities (…). Adding another pilot area at this stage would increase substantially the cost of the pilots, and would be impractical at this stage, without significantly increasing the depth or robustness of the evaluation” [40].

### Cluster sampling at the DWP

These two anecdotes can be interpreted in two opposite ways. One the one hand, they paint a flattering picture of the Department’s sampling decisions. On the other hand, they also reveal that the DWP has no set protocol for cluster sampling and that the procedure is, to a large extent, *negotiated*. Informal discussions with DWP officials confirmed that this was the case. They also confirmed the frequent use of *calls for expression of interest*, whereby JCP district managers are invited by the DWP to ‘bid’ to host new pilot programmes. Thus, there is some self-selection in the DWP’s cluster sampling decisions.

It is unclear from the above which of the two logics–policy commitments vs. research norms–is expected to have the strongest influence on cluster sampling decisions. However, these accounts lend credibility to the theory of CSB as the product of a cognitive dissonance between the two logics.

## Data and Methods

Although an experimental design would have allowed me to build a stronger causal theory, an observational design proved more feasible. In an ideal experiment, researchers would be randomly allocated to two or more groups. Groups would receive different endowments in terms of resources and information. As all research decisions are supposed to be documented in the relevant studies, reviews would be conducted, across all groups, before and after the intervention. To the extent that all groups are truly similar, any significant difference in the way clusters are sampled could be attributed to the intervention. In this study, the strategy is different: I estimated the probability of a given JCP district being selected as the pilot site in a given evaluation study, controlling for a number of covariates. The following section describes the PILOT-2 dataset that underpins this approach. All data and materials are available at https://osf.io/29d4s/.

### Selection process

**Fig 1:**
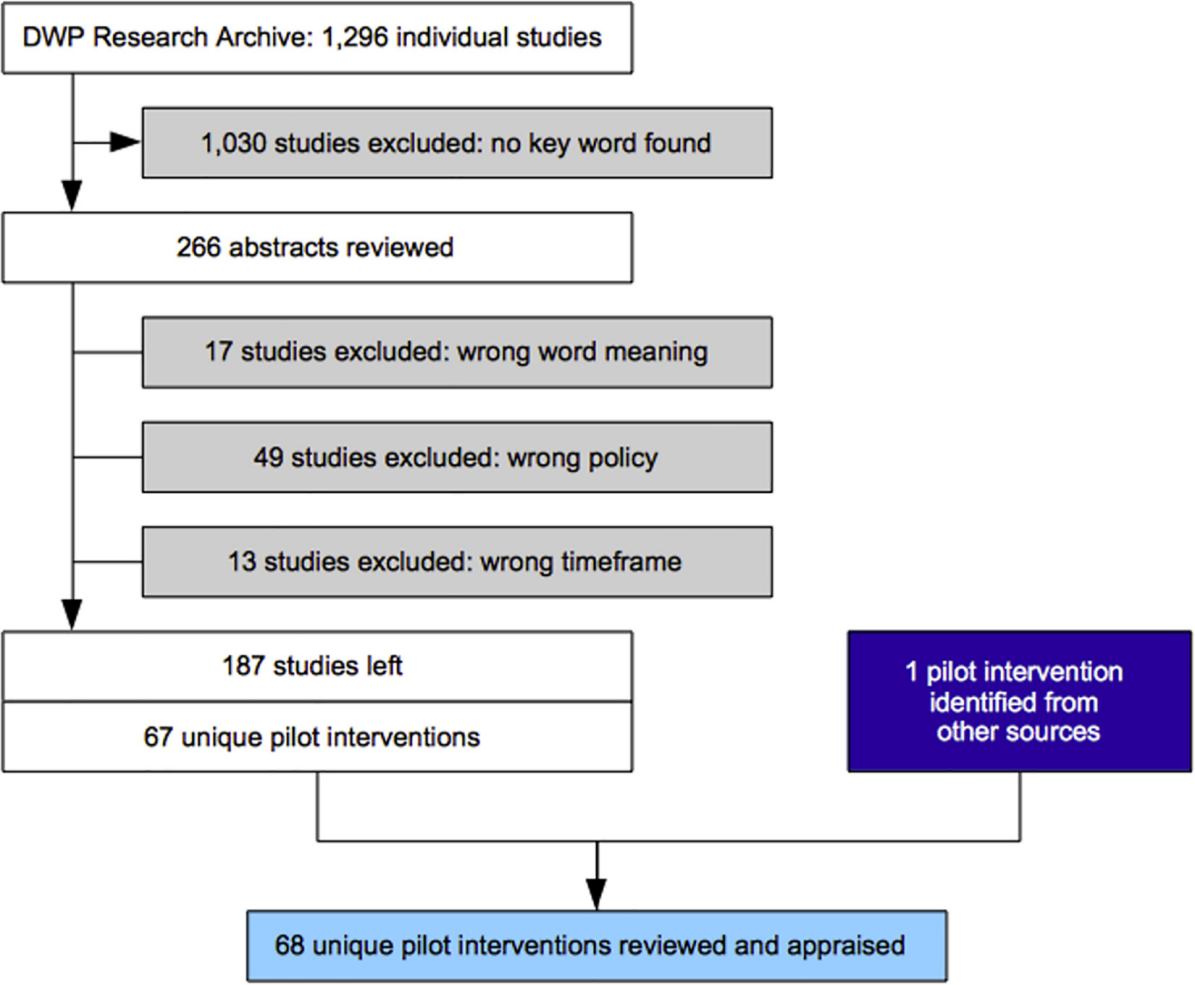
Selection process.

The selection process is shown in Fig 1. Studies were identified from the DWP Research and Statistics website which has since then been archived but remains accessible [41]. A standard web-scrapping programme was used to download the abstracts for all 1,296 available studies across four publication series: Research Reports (824 studies), In-House Reports (155 studies), Working Papers (106 studies) and Working Age and Employment Reports (211 studies). The abstracts were then screened using seven key words typically associated with pilot or experimental research in the UK [42]. These key words were: ‘pilot’, ‘trial’, ‘pathfinder’, ‘trailblazer’, ‘experiment’, ‘prototype’ and ‘demonstration’. All studies not mentioning one of these key words were excluded (1,030 studies). The abstracts of the 266 remaining studies were read and appraised. The study itself was only read when a decision could not be made based on the information provided in the abstract. Out of these 266 studies, I excluded 17 studies in which the key word was used with a different meaning (e.g. pilot questionnaire) or with reference to a study not commissioned by the DWP. I excluded another 49 studies that did not evaluate an active labour market policy as defined by the OECD [43]. Thus, for example, childcare and early education programmes were excluded, even though they sometimes have a positive effect on the labour supply. I excluded another 13 studies that were commissioned either before May 1997 or after May 2010. At this stage, 187 studies remained in the sample. The next step was to identify *unique pilot interventions*, given that (a) some interventions were subject to several evaluations (addressing different questions or taking place at different points in time) and (b) some studies evaluated several interventions. A total of 67 unique pilot interventions were identified (S1 Table).

Two additional channels were used to identify relevant pilot studies. Such precaution was meant to limit the risk of publication bias and to spot relevant studies which made no reference to one of the key words in their title or abstract. First, additional corporate and policy documents were processed in the exact same way as the DWP Research Archive. The reviewed documents include: (a) DWP annual activity reports (known as ‘Departmental Reports’) published between 2001 and 2010; and (b) four parliamentary research papers reviewing government-funded employment and training programmes [44]–[45]–[46]–[47]. Another unique pilot intervention was added to the dataset, bringing the total sample size to 68. The corresponding study was found in the DWP Research Archive and added to the corpus (188 studies). Second, a Freedom of Information Request was sent to the DWP on June 2012 to determine whether the publication of relevant studies may have been blocked. The department responded that this was not the case.

### Unit of analysis

Each new policy pilot required DWP researchers to sample a few clusters among the wider population of clusters, so each unit in my dataset is a ‘pilot-cluster’ combination (more specifically, as we will see later, a ‘pilot-district’ combination). As with the pilots, the identification of clusters presented a number of challenges. A first difficulty was that DWP uses three different networks to implement its labour market policies: JCP (which is part of DWP), local authorities and social service providers. Thus, the map of clusters was not identical across pilots. I chose the map of JCP districts as reference map, regardless of the network used for policy implementation, as this was a more common occurrence (probability = 0.68) than pilots delivered by providers (P = 0.26) or local authorities (P = 0.06). Another challenge was that the map of Jobcentre Plus districts changed several times during the observed period. In fact, Jobcentre Plus gradually replaced the Employment Service between October 2001 and April 2008. In addition, the map of JCP districts was revised on two occasions between 2001 and 2010. To ensure consistency, I used the 2010 map and its 40 districts (England only) as reference. Thus, for each of the 68 policy interventions piloted between May 1997 and May 2010, DWP officials had to sample a few districts from the larger pool of 40 JCP districts. This gives a total of 2,720 ‘pilot-district’ combinations.

I am confident that the above-mentioned assumptions did not significantly impact the validity of my analyses for several reasons. First, only one of the variables in the dataset is measured at the JCP district level; and it was analysed separately (relative performance). Other variables reflect either pilot characteristics (e.g. target group) or local labour market characteristics (e.g. population). Perhaps the most challenging implication of this assumption is that many variables had to be recoded from their original unit (usually local authorities) to JCP districts. This was done by means of a pool-up table matching each local authority with the corresponding JCP district (S2 Table).

The assumption that the 2010 map of JCP districts is representative of the network’s organisation for the whole period from 1997 and 2010 is admittedly more difficult to defend, but I believe that it is still a reasonable proxy. Indeed organisational changes have mainly concerned the Greater London area. Again, I conducted separate analyses omitting London when I thought this could cause a problem. Outside of London, the few changes to the map of JCP concerned the edges of the districts rather than their core. For example, the county of Rutland (population: 37,600 as of 2011) was separated from Lincolnshire (population: 714,000) and merged with Leicestershire and Northamptonshire (combined population: 1.02 million) in 2009.

### Variables

The dependent variable in this paper is a dummy indicating whether a given district *i* was selected as pilot site for a given intervention *j*. In the majority of cases, coding was straightforward. However, a judgment was required in two situations. The first situation occurred when there was only a partial correspondence between the district classification used in a given study and the classification used in the dataset. As already mentioned, a few interventions were piloted by external organisations, which were not required to provide services within a given JCP district. In these cases, the main locality where the service was provided was used to determine the pilot district. The second situation occurred when an intervention was piloted in just a few offices within a given JCP district. In this case, the district as a whole was considered as pilot site. It was indeed assumed that the link between the DWP and the different JCP offices was mediated by district managers. In other words, multistage sampling (with districts sampled first and offices subsequently) was considered more plausible than a direct sample of offices by the DWP. The high number of JCP offices across the UK and the hierarchical structure of JCP justified this decision.

The main independent variable is a measure of ‘absolute performance’, namely the rank of a given JCP district in terms of its capacity to move clients from ‘welfare to work’. JSA off-flow rates were used for that purpose, i.e. the proportion of Jobseeker Allowance (JSA) claimants moving into work in a given month [48]–[49]. This variable reflects the effectiveness of a district in matching the demand and the supply of labour, and is one of several performance indicators used by the UK government to monitor its employment programmes. The data was collected using DWP’s Stat-Xplore [50]. To reduce noise, the value included in the dataset is the annual average JSA off-flow rate of a given district the year before the launch of the pilot. Districts were then ranked from 1 (best outcome) to 40 (worst outcome).

Although readily available and regularly used by policymakers, the JSA off-flow data has limited construct validity. First, it is heavily influenced by local circumstances, such as the business cycle. Second, it does not take into account the expectations of the DWP and the fact that districts enjoying favourable labour market conditions will be assigned more ambitious performance targets. To address this concern, I included a second independent variable, namely a measure of the ‘relative performance’ JCP districts. This variable is based on the DWP’s ‘job outcome’ point system, which measures the number of JCP customers who move into work. When there is a match, the job outcome is converted into points depending on the customer group. The higher the priorities of the customer, the more points earned. For example, helping an unemployed lone parent into work earns a District 12 points, whereas helping an employed person change job earns only one point. Every year, new targets are established centrally by the DWP for each district based on previous performance and labour market circumstances. At the end of the year, a job outcome performance is measured in terms of percentage against target and a ranking of districts is established. Although it is much closer to the idea of performance than JSA off-flow rates, the job-outcome variable has two important limitations. First, district-level data is only available for 2007–2008. As a result, I only used it from pilots commencing between 2007 and 2009. Second, the district classification used by the DWP for its job outcome ranking differs from the classification used in my dataset. I dropped the values for which there was no match between the two classifications (10 districts out of the 40 for which I had performance data).

Moderating variables were also introduced. First, a dummy was created to distinguish ‘formal’ pilots from ‘pathfinder’ pilots. Pathfinders (also sometimes referred to as ‘trailblazers’ or ‘prototypes’) are pilots for which the government has publicly committed to roll out, regardless of the evaluation results. Conversely, one can never be sure whether a pilot will be rolled out or terminated. This variable, which was found in virtually all evaluation reports, was used as a proxy for the government’s commitment to the intervention.

The second moderating variable is a dummy indicating whether the intervention was delivered by the JCP network or by another organisation (local authority, professional service provider).

The control variables included in this paper are the sampling variables identified in the evaluation reports. First, JCP districts were coded as belonging to one of four great regions. The 11 districts in the North East of England, North West of England and Yorkshire and the Humber were coded as belonging to the ‘North of England’. The eight districts in the East and West Midlands were merged into the ‘Midlands’ region. The nine London districts were coded as such. The 12 districts in the East of England, South West and South East were coded as being part of the ‘South of England’.

The proportion of benefit claimants in a given JCP district was estimated using the ratio of all claims (Jobseeker Allowance, Income Support and Incapacity Benefit) in the active population of this district in August 2007 (Source: Nomis database) [51]. The data was found to be fairly representative of the entire period.

The working age population of each district is the number of individuals aged 16 to 59 (women) or 64 (men) in mid-2003. The data was provided by the Office for National Statistics (ONS) [52].

The population density of each district is the estimated resident population in mid-2010 (source: ONS) divided by its size in hectares (source: ONS) [52].

The cumulated number of pilots in a given district is the number of pilots hosted by a district between May 1997 and the pilot under consideration.

Additional variables were included in the dataset at a later stage to explore possible sub-group effects. Those include: (i) the proportion of individuals not identifying themselves as ‘white’ in the adult population in 2007, (ii) the proportion of lone parents claiming income support in the working age population 2003, and (iii) the proportion of Incapacity Benefit (IB) claimants in the working age population in 2003. All these figures were found on the NOMIS and ONS websites.

Programmes targeting (i) ethnic minorities, (ii) lone parents, and (iii) disabled people were identified based on information found in evaluation reports.

The full PILOT-2 dataset is attached to this paper (S3 Table). Descriptive statistics can be found in Table 1.

**Table 1:** Descriptive statistics.

Variable	N	Min	Max	Mean	SD	Freq (1)
Effective pilot site	2,600	0	1	--	--	533
Region–North	2,720	0	1	--	--	748
Region–Midlands	2,720	0	1	--	--	544
Region–London	2,720	0	1	--	--	612
Region–South	2,720	0	1	--	--	816
Pathfinder	2,720	0	1	--	--	400
JCP-led programme	2,720	0	1	--	--	1,880
Programme targeting ethnic minorities	2,720	0	1	--	--	160
Programme targeting lone parents	2,720	0	1	--	--	400
Programme targeting disabled people	2,720	0	1	--	--	440
Absolute performance (rank)	2,560	1	40	20.5	11.54	--
Relative performance (rank)	360	1	47	22.26	14.43	--
Benefit claimants (%)	2,720	1.3	7.3	3.08	1.42	--
Working age population (in 100,000)	2,720	3.98	15.37	7.71	2.75	--
Population per ha (in 10)	2,720	0.1	26.8	2.92	5.24	--
Cumulated number of pilots	2,600	0	25	6.35	4.82	--
Ethnic minorities (%)	2,720	4	43	15	10.44	--
Lone parents claiming Income Support (%)	2,720	0.3	5.76	2.48	1.27	--
Incapacity Benefit claimants (%)	2,720	2.33	23.75	8.55	5.34	--

## Results

### Were clusters selected with a view to maximise generalisability?

**Fig 2:**
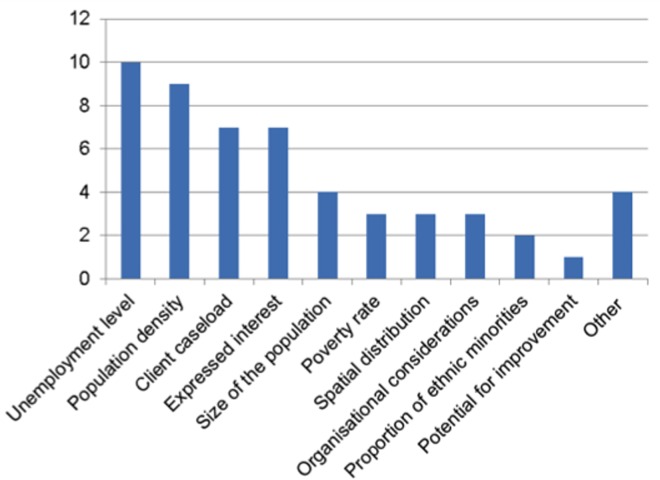
Frequency distribution of cluster sampling variables used in DWP pilots.

The review of evaluation reports leading to the development of the dataset showed that the reporting of cluster sampling decisions was highly inconsistent across studies and poor on average. The most commonly reported information was the number of pilot districts (reported in 65 studies out of 68; probability = 0.96) and the location of these districts (reported in 56 studies out of 68, P = 0.82). The protocols followed to get to these samples are much less transparent. No flow diagram was provided (P = 0). Sampling variables were reported in 28 studies (P = 0.41). Sampling methods were reported in 20 studies (P = 0.29). The representativeness of the selected pilot districts was discussed in 13 studies (P = 0.19), only briefly in most cases. On average, a new policy intervention was piloted in 8.2 JCP districts, i.e. a fifth of the territory. The four most frequent sampling variables were the level of unemployment in each cluster (10 counts), the population density (C = 9), the size of the client caseload (C = 7) and whether district managers expressed their interest in piloting the intervention, usually through a formal bidding process (C = 7). Fig 2 shows a frequency distribution of this variable.

### Were ‘effect-enhancing’ clusters more likely to be sampled?

**Fig 3:**
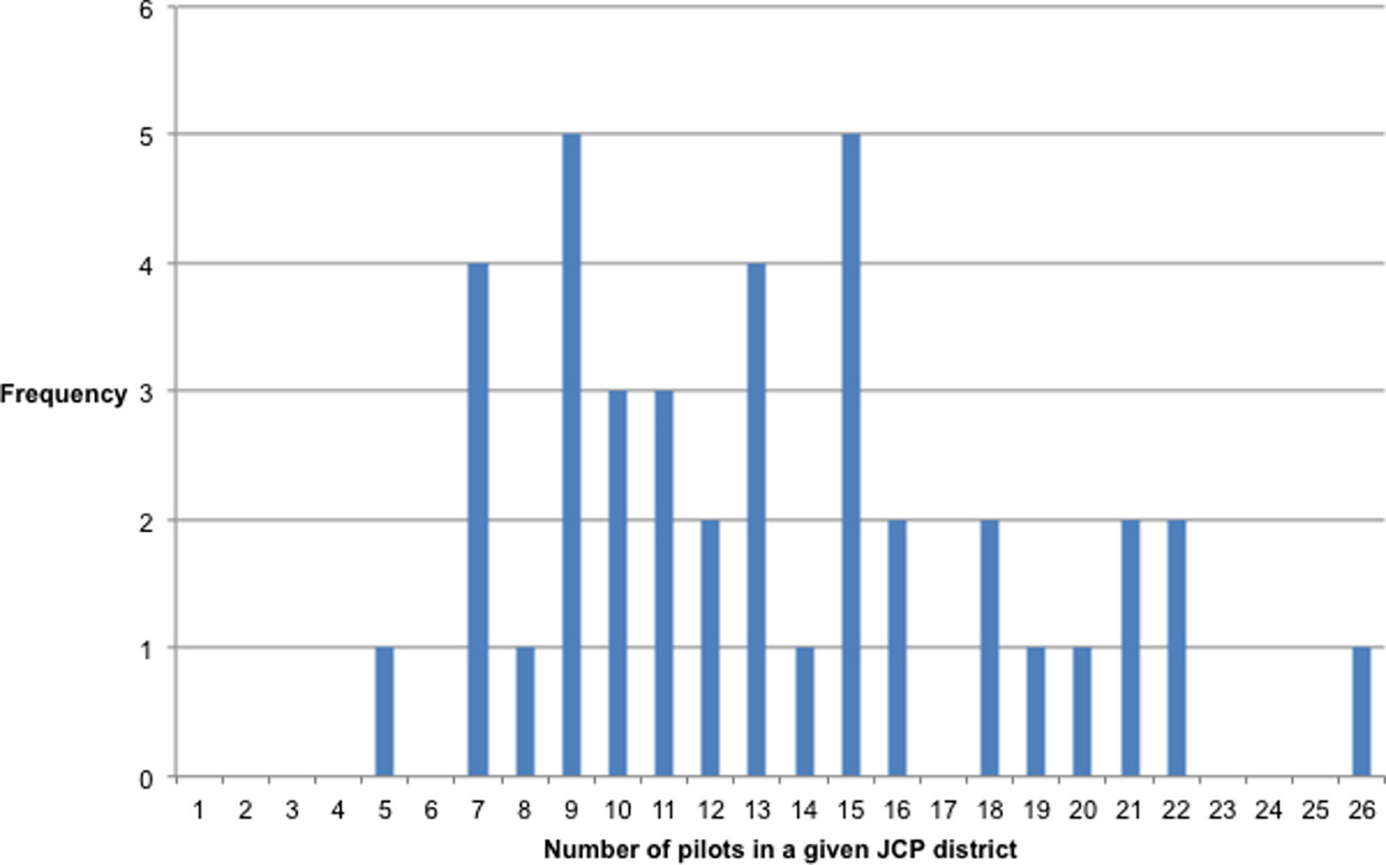
Frequency distribution of the number of pilots per JCP district.

It is useful to recall that the dataset used in this paper includes 68 policy interventions. Each new policy pilot required DWP officials to sample a few districts from a wider pool of 40 JCP districts. This means that each unit in the dataset is one of the 2,720 possible ‘pilot-district’ combinations. However, it was not possible to identify the pilot districts selected for three of the 68 pilots, which means that the analyses discussed below are based on 2,600 known ‘pilot-district’ combinations. Out of those, there are 533 ‘effective’ pilot-districts, i.e. districts which were effectively selected to run a pilot intervention. We can thus infer that between May 1997 and May 2010, an average JCP district was sampled about 13 times. Hampshire and the Isle of Wight had the lowest sampling frequency (5 occurrences). Birmingham and Solihull was the most sampled district (26 occurrences). A frequency distribution can be found in Fig 3.

A series of binary logistic regressions were used to model the odds of being selected as pilot district vs. not (using maximum likelihood estimation). Three models are presented in Table 2. Model 1 tests the partial effect of local labour market conditions on the odds that a district will be sampled vs. not, controlling for a number of variables. Model 2 tests the effect of district performance on the odds that a district will be sampled vs. not, controlling for the same variables. Model 3 is a more parsimonious proposition.

**Table 2:** Odds ratio of being selected as pilot district vs. not (models 1 to 3).

	(1)	(2)	(3)
Benefit claimants (%)	1.12**	1.30*	1.13***
Working age population (in 100,000)	1.05**	1.1	1.06**
Population per ha (in 10)	1.03**	1.06	1.04***
Region–Midlands	0.97	1.5	--
Region–London	0.69*	0.61	0.67**
Region–South	0.57***	0.62	0.60***
Cumulated number of pilots	1.02	0.96	--
Absolute performance (rank)	1.01	--	--
Relative performance (rank)	--	1.01	--
Intercept	0.1	21.8	0.13
N	2,400	330	2,600

- Binary logistic regression

- Y = PILOT

- Coefficients are odds ratios

* *p<0*.*1*

** *p<0*.*05*

***p<0.*01*

The results show no significant association between the ‘absolute performance’ of a district and the odds of being selected as pilot site. Controlling for the proportion of benefit claimants, the size of the working age population, the population density, the region and the number of pilots already run in the district since 1997, a one-place fall in the ranking of districts in terms of JSA off-flow increased the odds of a given JCP district to be sampled by about 1%. This effect is not significant at the 5% level.

Likewise, no significant association was found between the ‘relative performance’ of a given JCP district and the odds of being sampled vs. not. Controlling for the above-mentioned variables, a one-place fall in the ranking of districts in terms of performance increased the odds of a given JCP district to be sampled by about 1%. This effect is not significant at the 5% level.

The results in Table 3 show that the effect of effect-enhancing conditions (be it in terms of absolute or relative performance) was not stronger when the government was publicly committed to the reform, as hypothesised (see models 4 and 6). For example, controlling for other variables, a one-place fall in the ranking of districts in terms of JSA off-flow increased the odds of a given JCP district to be sampled by about 1% when the government was not committed to the reform, and decreased it by about 2% when the government was committed (model 4). The interaction is not significant.

**Table 3:** Odds ratio of being selected as pilot district vs. not (models 4 to 7).

	(4)	(5)	(6)	(7)
Benefit claimants (%)	1.12**	1.11**	1.23*	1.22*
Working age population (in 100,000)	1.06**	1.06**	1.06	1.06
Population per ha (in 10)	1.03**	1.03**	1.06	1.06
Region–London	0.64**	0.65**	0.50	0.51
Region–South	0.53***	0.53***	0.56	0.56
A. Absolute performance (rank)	1.01*	1.02*	--	--
B. Relative performance (rank)	--	--	1.01	1.00
C. Pathfinder	0.89	--	0.26	--
D. JCP-led programme	--	1.25	--	0.41
Interaction A*C	0.98	--	--	--
Interaction A*D	--	0.99	--	--
Interaction B*C	--	--	0.99	--
Interaction B*D	--	--	--	1.01
Intercept	0.11	0.09	0.08	0.10
N	2,520	2,520	330	330

- Binary logistic regression

- Y = PILOT

- Coefficients are odds ratios

* *p<0*.*1*

** *p<0*.*05*

***p<0.*01*

The effect of effect-enhancing conditions was not stronger when the intervention was implemented by JCP, as opposed to local authorities or non-governmental services providers (see models 5 and 7). For example, a one-place fall in the ranking of districts in terms of performance had absolutely no effect on the odds of being sampled when the intervention was not implemented by JCP and increased the odds of being sampled by 1% when the intervention was implemented by JCP (model 7). The interaction is not significant.

### Did all sites have the same probability of being sampled?

Controlling for other variables, districts with a greater proportion of benefit claimants were more likely to be sampled. Indeed, for each additional percentage point increase in the proportion of benefit claimants, the odds of a district being sampled increased by between 11% and 30% depending on the specifications. The effect is significant at the 5% level in four out of seven models (models 1, 3, 4 and 5).

Controlling for other variables, districts with larger working age populations were more likely to be sampled. Indeed, for each additional 100,000 people in the working population, the odds of a region being selected increased by 5% and 10% depending on the specifications. The effect is significant at the 5% level in four out of seven models (models 1, 3, 4 and 5).

Controlling for other variables, districts with higher population density had a greater chance of being sampled. On average, an increment of 10 people per hectare in a given district increased the odds of this district being selected by between 3% and 6%. This result is significant at the 5% level in four out of seven models (models 1, 3, 4 and 5).

Controlling for other variables, districts in the North of England and the Midlands were more likely to be selected as pilot sites than districts in any other part of the country. For example, the odds of a southern district to be selected as pilot site were, depending on the specifications, between 38% and 47% lower than for a northern district, controlling for other variables. This cannot be explained solely by the respective size of each region. To understand this result, it is important to remind the reader that the North of England comprises 11 districts, the Midlands 8 districts, London 9 districts and the South 12 districts. If one district from each region was selected as pilot–as implied by some evaluation reports, a given southern district would have an 8% chance of being selected and a northern district a 9% chance. If my assumption was true, the odds of a southern district to be selected as pilot site would be expected to be 0.92 those of a northern district, *i*.*e*. only 8% lower (as opposed to between 38% and 47% lower). More strikingly, the odds of a London district would be expected to be 1.22 those of a northern district, *i*.*e*. 22% *higher*.

Controlling for other variables, districts which had hosted a high number of pilots since May 1997 were not less likely to be sampled. Indeed, for each additional pilot run in a district since 1997, the odds of seeing this district being sampled again varied from between +2% and -4% depending on the specification. These effects are not significant.

### Did a high client caseload make a district more likely to be sampled?

Exploratory sub-group analyses were conducted to further investigate the influence of the client caseload on the odds that a given district would be sampled vs. not. Between 1997 and 2010, there were indeed three main welfare benefits in the UK, targeting different groups with different problems. Jobseeker Allowance (JSA) was the main unemployment insurance. Income Support (IS) was an income supplement for people on low income but unable or not expected to actively look for work, such as lone parents. Incapacity Benefit (IB), later replaced by the Employment and Maintenance Allowance, was paid to active people who were unable to work because of illness or disability. In addition, some interventions were primarily intended to ethnic minorities, whether they were benefit claimants or not.

Table 4 presents the results of a series of interactions for three specific client groups: ethnic minorities, lone parents claiming IS and IB claimants.

**Table 4:** Odds ratio of being selected as pilot district vs. not (model 8).

	(8)
Population per ha (in 10)	1.04***
Region–London	0.36***
Region–South	0.50***
E. Ethnic minorities (%)	1.01**
F. Programme targeting ethnic minorities	0.15***
Interaction E*F	1.13***
G. Lone parents claiming Income Support (%)	0.95
H. Programme targeting lone parents	0.37***
Interaction G*H	1.42***
I. Incapacity Benefit claimants (%)	0.99
J. Programme targeting disabled people	2.31**
Interaction I*J	0.99
Intercept	0.27
N	2,600

- Binary logistic regression

- Y = PILOT

- Coefficients are odds ratios

** *p<0*.*05*

***p<0.*01*

Controlling for other variables, a one-percentage point increase in the proportion of ethnic minorities living in a given JCP district increased the odds that this district would be sampled by 1% when the intervention did not focus on ethnic minorities. However, it increased the odds by 13% when the intervention did focus on ethnic minority. The interaction was found to be strongly significant.

Each additional percentage point in the proportion of lone parents claiming IS in a given JCP district decreased the odds that this district would be sampled by 5% when the intervention did not primarily target lone parents. However, it increased the odds by about 42% when the intervention targeted lone parents.

Each additional percentage point in the proportion of IB claimants in a given JCP district decreased the odds that this district would be sampled by 1% when the intervention did not primarily target disabled people. The exact same effect was observed for interventions targeting disabled people. Unsurprisingly, the interaction is not significant.

The table also confirms previous results. It shows that the population density remains a stronger predictor of whether a JCP district will be chosen as pilot site. Likewise, the geographic disparity among regions–already observed in Table 3 –remains, with London and the South of England less likely to be sampled than the North of England and the Midlands.

## Discussion

The systematic review of the evaluation studies commissioned by the DWP highlights four important lessons. First, evaluators rarely reflect on the generalisability of their findings. This low level of concern can be seen in the inconsistent, and generally poor, reporting of essential research decisions. For example, sampling variables were reported in about 4 studies out of 10. This finding is in line with previous studies in the area of clinical trials, which have also highlighted the lack of transparency of sampling decisions [10]–[15]–[53]–[54]–[55]. The lack of consideration for external validity becomes more obvious when one considers the sampling variables and criteria that are actually reported. This study confirms the *Standard Model of impact evaluation* developed by Orr [3], whereby policy pilots and experiments are based on a small number of purposively selected sites. It also gives credibility to the idea that the prime sampling method used in these studies is often *stratified convenience sample* [4]. In this model, investigators pre-select clusters that are representative of the territory (e.g. x urban clusters, y rural clusters) but given the constraints, eventually include the most convenient clusters (e.g. those that were easiest to persuade).

Second, ‘effect-enhancing’ districts were not more likely to be sampled. This finding might contrast with previous studies [10]–[25]; however it takes a more systematic approach and considers a much broader range of cases. The lack of association was found robust across models, across service providers (JCP vs. other providers) and regardless of whether the government was committed to the intervention or not. The construct validity of the two variables capturing the ‘enhancing’ effect of some JCP districts can be demonstrated, given that they both derive from indicators provided, and routinely used, by the DWP and JCP to manage their programmes.

The third finding is that policy interventions were often piloted in the districts where the proportion of client groups among the working population was the highest. For example, interventions targeting ethnic minorities tended to be piloted in JCP districts where ethnic minorities were relatively more numerous. Conversely, interventions targeting other groups were piloted in districts where the proportion of ethnic minorities was closer to the national mean. The same result was found with interventions targeting lone parents. However, there was one notable exception: the proportion of IB claimants did not seem to significantly influence the sampling of JCP districts, whether or not the intervention was intended for disabled people. This association can be interpreted in three different ways. First, selecting clusters that offer the largest study samples could be a way of reducing the costs of the evaluation [4]. Second, it could be that researchers anticipating a small policy effect tried to capture this effect by multiplying the number of statistical tests, which is easier when the sample is large. This practice is known as ‘p-hacking’ (or data fishing, data snooping, equation fitting and data mining). Evidence suggests that p-hacking is widespread throughout science, although its effect seems to be weak relative to the real effect sizes being measured [56]. Third, it could be that researchers expecting a positive policy effect tried to implement the policy where it was most needed. This is congruent with the idea that agents can be driven by a desire to maximise their moral reputation [57]. All three theories indicate the presence of confirmation bias, which is the tendency to search for, interpret, or recall information in a way that confirms one's beliefs or hypotheses [58].

The fourth and last finding from this study is that some JCP districts were consistently oversampled. This includes districts from the North of England and the Midlands and more densely populated districts. This effect is robust across all models and even when the proportion of benefit claimants is controlled for. This result is surprising. A possible explanation is that new interventions tended to be piloted in Labour constituencies outside London. Indeed, the map of pilots seems to match to a large extent the map of Labour constituencies. Given the symbolic property of pilot programmes [59]–[60], one could argue that pilots are used to give a distributive advantage to some regions, through an early access to new programmes and budgets.

Unfortunately, it is difficult to analyse the extent to which these findings match the descriptions of the DWP reviewed in section 3. Indeed, these descriptions are too general to give any reliable insight on cluster sampling decisions.

## Implications

The results of my systematic review of government-sponsored evaluations in the UK have implications beyond the case itself. This concluding section looks at these implications from theoretical, methodological and professional perspectives.

### Theoretical implications

This study was based on the premise that CSB is more than just a statistical property that can be prevented or corrected with statistical tools, as assumed in the rest of the literature. It is also an individual and social behaviour that can be ‘nudged’, provided it is well understood. Thanks to the PILOT-2 dataset, we now have a better understanding of the ways in which CSB manifests itself. The strong and persistent correlation between a district’s client caseload and its probability of being selected as pilot site suggests that this effect is more subtle and indirect than previously thought. In particular, the selection of high-volume districts entails a risk of confirmation bias, which warrants further investigation. Conversely, the hypothesis that CSB would directly lead to the oversampling of effect-enhancing clusters can clearly be rejected. Although, this is a very narrow definition of an effect-enhancing cluster. An analysis of the influence of local policy entrepreneurs, including JCP district managers and local members of parliament, would be a welcome addition.

Unfortunately, the design of the study did not allow the identification of the cognitive and social mechanisms that trigger CSB. Nevertheless, it sheds light on the factors that make CSB more likely to appear. One factor is the relatively low salience of the issues of external validity and sampling bias among policy evaluators, as suggested by the poor reporting of these important research decisions. The most likely explanation is that the profession’s heightened focus on the question of internal validity over the past decade has *de facto* put the problem of external validity on the back seat. Such hypothesis remains to be fully tested. Conversely, the government’s commitment to a reform, which was thought to increase researchers’ cognitive dissonance, did not seem to influence the selection of JCP districts.

### Methodological implications

The empirical strategy devised to address the question of CSB, combining a narrow scope with a deep focus, proved to be a winning one. In particular, it allowed integrating three types of variables–study-specific variables, policy-specific variables and area-specific variables–into a single dataset. I hope that the substantive credibility of this study’s conclusions will stimulate further uses of PILOT-2 or similar datasets.

Obviously, this approach is not without flaws. Researchers wishing to contribute to the CSB literature are advised to fully exploit the following limitations. First, the data was collected and coded by a single person. To limit measurement error, double coding should be employed. Second, the recoding of many variables from a ‘local authority’ basis to a ‘JCP district’ proved very labour-intensive. Colleagues are advised to use clusters in a way that minimises such recoding and data collection. Third, this study focused on the sites that were selected for a pilot. However, we know of cases where some sites agreed to participate in a pilot and subsequently refused to participate in the evaluation. Thus, a focus on sites participating in the evaluation is needed. Fourth, the design of this paper means that its findings can be generalised to the devolved nations of the UK (Northern Ireland, Scotland and Wales), which all share a large number of government agencies with England, like JCP. However, a generalisation beyond the UK would be perilous. Data from other countries and policy areas would be very helpful.

### Professional implications

At best, evaluators failing to address CSB in their research leave policy-makers, meta-analysts and other stakeholders with the difficult task of making guesses regarding the generalisability of their conclusions. At worst, they mislead them about the true effect of the intervention on the population of interest. Peer reviewers and research commissioners are advised to be more demanding in the way sampling decisions are discussed and reported [61]. Furthermore, investigators are advised to take the following steps (largely based on Larry Orr’s own recommendations) [3]:

1. Designate studies as either efficacy or effectiveness studies;
2. Define the population of policy interest at the outset;
3. Think about how you can select sites and draw samples that have a reasonable relationship to that population of interest;
4. Acknowledge constraints such as costs;
5. Compare your sample to the population of policy interest on relevant characteristics and outcomes;
6. Once you have results, use one of the various techniques that are available to project your estimates to the population of policy interest;
7. Triangulate findings with case studies and interviews of service providers;
8. Report those results along with the results for your actual sites using guidelines such as CONSORT or STROBE.

## Supporting Information

S1 Table Sift process. (XLSX)

S2 Table Look-up table. (XLSX)

S3 Table PILOT-2 Dataset. (XLSX)
